# Evaluation of Systemic Injury in Calves with Rotavirus-Induced Diarrhea Using Sensitive Biomarkers and Immunopathology

**DOI:** 10.3390/ijms27010065

**Published:** 2025-12-20

**Authors:** Murat Uztimür, Cennet Nur Ünal, Muhammet Bahaddin Dörtbudak, Davide Bisanti, Alessandro Di Cerbo

**Affiliations:** 1 Faculty of Veterinary Medicine, Department of Internal Medicine, Bingöl University, Bingol 12200, Türkiye; muratuztimur@yahoo.com (M.U.); cnaltunboga@gmail.com (C.N.Ü.); 2 Faculty of Veterinary Medicine, Department of Pathology, Harran University, Şanlıurfa 63290, Türkiye; mbdortbudak@gmail.com; 3 San Francesco Veterinary Clinic, 73016 San Cesario di Lecce, Italy; 4 School of Biosciences and Veterinary Medicine, University of Camerino, 62024 Matelica, Italy

**Keywords:** calf, lung, liver, rotavirus, biomarker, histopathological, immunohistochemical

## Abstract

Studies in human medicine have demonstrated that rotavirus infection can also affect extraintestinal sites due to its systemic effects. However, in veterinary medicine, the injury caused by rotavirus diarrhea is limited to the intestines, and its effects on various systemic structures remain poorly understood. In this observational case–control study, we aimed to determine the effects of HSP-27, Caspase-3, IL-2, γ-H2AX, HMGB-1, SP-D, and GDH (or GLDH) on the pathogenesis of rotavirus infection by using biomarkers for diagnostic purposes in lung and liver injury in neonate diarrheic calves naturally infected with rotavirus, both alive and post-mortem. Fifty-two Simmental calves (1–28 days old) of both sexes, 40 infected with rotavirus and 12 healthy, were studied. Twenty-eight out of 40 survived, while the remainder underwent necropsy for histopathological and immunopathological (HSP-27, Caspase-3, IL-2, γ-H2AX) examination of the lungs and livers. Lung and liver-specific serum E-selectin, glutamate dehydrogenase, surfactant protein-D, and high mobility group box-1 were analyzed by a bovine-specific ELISA kit (Shanghai Coon Koon Biotech Co., Ltd., China). Histopathological and immunohistochemical analyses confirmed lung and liver injury in naturally infected calves. HMGB-1, SP-D, and GDH concentrations were significantly higher in naturally infected calves than in the control group (*p* < 0.001, *p* < 0.001, and *p* < 0.05, respectively), showing an excellent diagnostic predictive capacity for lung and liver injury. Also, IL-2, HSP-27, CASP-3, and γ-H2AX were significantly expressed in the lungs (*p* < 0.001, *p* < 0.001, *p* < 0.001, and *p* < 0.05, respectively) and liver (*p* < 0.001, *p* < 0.001, *p* < 0.01, and *p* < 0.01, respectively). All these observations led us to hypothesize that oxidative stress, apoptosis, and DNA damage may underlie the pathogenesis of this condition. Nevertheless, further studies on large populations of rotavirus-infected calves are needed to confirm the data reported in the current study.

## 1. Introduction

Neonatal calf diarrhea is a disease with high morbidity and mortality, has a multifactorial etiology, and is commonly observed in calves with an immature immune system [1,2]. Losses resulting from calf diarrhea can be minimized by treating the bacterial and parasitic agents that cause the disease, identifying the injury they cause to various organs, and detecting this injury early [3,4,5]. However, no studies have yet been conducted on the treatment of rotavirus, the early detection of the injury it causes to various organs, or the pathogenesis of this organ injury. Understanding the injury caused by rotavirus in the lungs and liver through various pathological techniques and organ-specific biomarkers enables early diagnosis, clarification of its pathogenesis, and the implementation of therapeutic interventions.

High mobility group box (HMGB-1) is a member of a family of proinflammatory cytokines released from injured tissues and is also involved in transcription and gene regulation [6]. This protein acts as a signal for the activation and chemotaxis of inflammation-mediated immune cells such as neutrophils, monocytes, and macrophages [7,8]. Its overexpression leads to tissue and organ failure by triggering an uncontrolled immune response [6,9]. Studies on many diseases, including sepsis, cardiovascular shock, diabetes, and cancer, have reported that HMGB-1 concentrations vary significantly and have a significant impact on their pathogenesis [6,8,10]. Based on this, researchers have used HMGB-1 inhibitors therapeutically in clinical practice and achieved effective results [10,11,12].

On the other hand, surfactant Protein D (SP-D), a member of the collectin family produced by alveolar type 2 and club cells, plays a pivotal role in regulating the innate immune system in the lungs [13]. In recent years, many studies have reported that SP-D concentration can serve as a non-invasive diagnostic and prognostic biomarker for respiratory diseases [14,15,16,17]. In addition, intratracheal administration of SP-D has been shown to significantly inhibit lung inflammation in mice with experimentally induced pulmonary injury [18].

Glutamate dehydrogenase (GDH or GLDH) is an enzyme found in the mitochondrial matrix and, to a lesser extent, in the endoplasmic reticulum. It is expressed in the central periphery of the liver and has been reported to be significantly deregulated in mitochondrial dysfunction and hepatic necrosis [19,20]. Furthermore, recent studies have indicated that GDH is much more sensitive and specific than alanine aminotransferase (ALT) in detecting liver injury [21,22]. Therefore, early detection of liver injury using GDH is crucial, as it enables rapid and effective therapeutic intervention.

Viral infections can lead to a range of pathological conditions, including DNA damage, oxidative stress, apoptosis, and inflammation [23,24,25,26]. Thorough demonstration of these pathological disorders significantly contributes to understanding disease pathogenesis. Recent studies have reported that the γ-phosphorylated form of histone *H2AX* (γ-H2AX) is effective in sensitively detecting DNA breaks, caspase-3 in detecting apoptosis, interleukin-2 (IL-2) in inflammation, and heat shock protein-27 in oxidative stress [27,28,29,30]. γ-H2AX, formed by phosphorylation of histone *H2AX*, is widely used as a biomarker for DNA damage [26]. This biomarker has been reported to be significantly expressed in the liver of patients with hepatocellular carcinoma and can be used to predict disease progression [31]. IL-2 is a proinflammatory cytokine produced by CD4^+^ T lymphocytes and various other immune system cells, and its levels in tissue are significantly elevated in many diseases [25]. Caspase-3 (CASP-3) is a key mediator of apoptosis in vivo and is activated by intrinsic and extrinsic pathways [24]. It has been reported that CASP-3 expression can be used to demonstrate hepatic apoptosis in experimentally induced pneumonia caused by *Escherichia coli* [32]. Heat shock protein-27 (HSP-27) is a marker of oxidative stress. In a study by King et al. (2000), HSP-27 expression was identified as a strong prognostic indicator of survival in patients with hepatocellular carcinoma [23].

This study was designed to test the hypothesis that rotavirus may cause lung and liver injury in calves with neonatal diarrhea, and that this injury could be assessed using different biomarkers. It also evaluated the diagnostic and prognostic utility of lung- and liver-specific biomarkers for determining in vivo injury to these organs in calves naturally infected with rotavirus, and to demonstrate lung and liver tissue injury in non-surviving calves using histopathological and immunopathological techniques.

## 2. Results

### 2.1. Biochemical, Biomarker, and Clinical Examination Findings

Clinical examination findings and the mean, standard deviation, minimum, and maximum values of biomarker and biochemical concentrations in rotavirus-infected calves are presented in Table 1.

White blood cells (WBCs) and potassium concentrations in rotavirus-infected calves were significantly higher than those in the control group (*p* < 0.001 and *p* < 0.05, respectively). Blood pH, calcium, bicarbonate, and glucose concentrations were significantly lower in rotavirus-infected calves than in the control group (*p* < 0.001, *p* < 0.001, *p* < 0.001, and *p* < 0.05, respectively). In contrast, there were no statistically significant differences between the rotavirus and control groups in heart rate, body temperature, hematocrit (HCT), sodium, chloride, total protein, albumin, BUN, or creatinine concentrations.

Serum biomarker concentrations in rotavirus-infected calves are presented in Table 2.

HMGB-1, SP-D, and GDH concentrations were significantly higher in calves naturally infected with rotavirus than in the control group (*p* < 0.001, *p* < 0.001, and *p* < 0.05, respectively). However, no statistically significant difference was observed between the rotavirus and control groups for E-selectin.

### 2.2. The Receiver-Operating Characteristic Curve (ROC) Analysis of Lung and Liver-Specific Biomarkers

The diagnostic significance of lung and liver biomarkers in healthy and neonatal calves is presented in Table 3.

**Figure 1:**
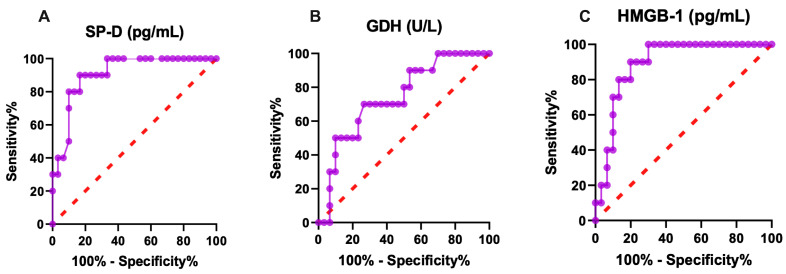
Graphical representation of diagnostic (**A**) SP-D, (**B**) GDH, (**C**) HMGB-1 ROC analysis of lung and liver injury in rotavirus-infected calves. SP-D: surfactant protein-D, GDH: glutamate dehydrogenase, HMGB-1: high mobility group box.

In ROC analysis, SPD had an AUC of 0.91, a sensitivity of 80%, a specificity of 93%, and a cut-off value of 1.955 pg/mL. HMGB-1 had an AUC of 0.89, with a sensitivity of 80%, a specificity of 80%, and a cut-off value of 518.8 pg/mL. These results indicate that SP-D and HMGB-1 exhibit excellent diagnostic performance for detecting lung and liver injury. GDH had an AUC of 0.74, a sensitivity of 70%, a specificity of 70%, and a cut-off value of 1.185 U/L. SP-D, GDH and HMGB-1 concentrations of calves naturally infected with rotavirus are shown in Figure 1, demonstrating moderate diagnostic performance compared with the excellent performance of SP-D and HMGB-1. In addition, SP-D and HMGB-1 showed excellent diagnostic value for assessing lung and liver injury, whereas GDH showed moderate diagnostic performance in calves naturally infected with rotavirus. Taken together, these biomarkers can be used to evaluate lung and liver injury.

### 2.3. Correlation Analysis

**Figure 2:**
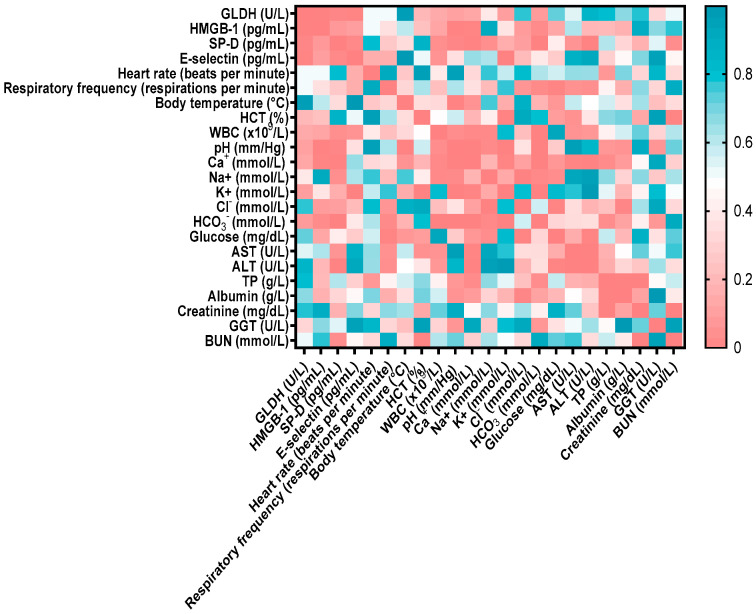
Correlation matrix between biochemical parameters, clinical examination findings, and lung and liver biomarkers. The color scale reflects the correlation coefficient: red indicates positive correlations, and blue indicates negative correlations. The strength of the correlation varies with color intensity. HMGB-1: high mobility group box, SP-D: surfactant protein-D, GLDH: glutamate dehydrogenase, WBC: white blood cells, ALT: alanine amino transferase, GGT: gamma-glutamyl transferase, BUN: blood urea nitrogen, HCT: hematocrit, AST: aspartate aminotransferase.

GDH showed a significant positive correlation with HMGB (r = 0.533, *p* < 0.001), SP-D (r = 0.364, *p* < 0.05), and E-selectin (r = 0.398, *p* < 0.05). HMGB-1 exhibited significant negative correlations with blood pH (r = −0.434, *p* < 0.05), calcium (r = −0.407, *p* < 0.01), and bicarbonate (r = −0.332, *p* < 0.01). Additionally, SP-D showed positive correlations with E-selectin (r = 0.338, *p* < 0.05), ALT (r = 0.432, *p* < 0.05), and blood urea nitrogen (BUN) (r = 0.313, *p* < 0.05), and negative correlations with blood pH (r = −0.378, *p* < 0.05), calcium (r = −0.428, *p* < 0.01), and bicarbonate (r = −0.490, *p* < 0.001). The detailed relationships between biochemical parameters and lung and liver biomarkers are illustrated in the heatmap in Figure 2. The positive correlations of GDH with other parameters indicate that as liver injury increases, concentrations of E-selectin, HMGB-1, and SP-D also increase. Conversely, the negative correlations suggest that parameters such as blood pH increase significantly as HMGB-1 concentration decreases.

### 2.4. Histopathological Findings

**Figure 3:**
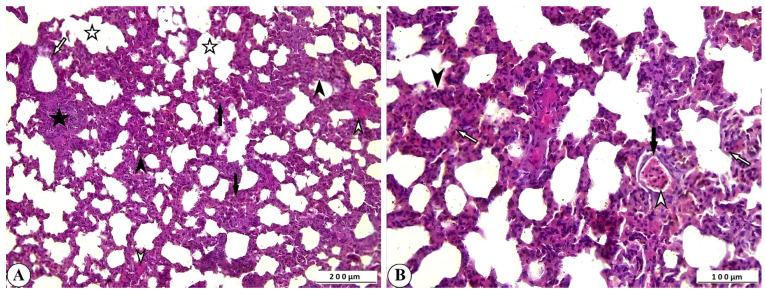
(**A**) Inflammatory cell infiltration and interalveolar thickening due to proliferation (arrowheads), peribronchiolar lymphoid hyperplasia (star), fibromuscular hypertrophy (open arrowheads), degenerative-necrotic changes in bronchiolar epithelium (open arrow), alveolar emphysema (open stars), and atelectasis (arrows) in cattle lung, ×100, Bar: 200 µm, HE. (**B**) Interalveolar thickening (arrowhead), degenerative-necrotic changes in bronchiolar epithelium (open arrow), inflammatory cell infiltration (open arrowhead), hyperemia (arrow) in cattle lung, ×200, Bar: 100 µm, HE.

Histopathological examination of lung tissue from rotavirus-infected calves generally revealed a pattern with interstitial pneumonia. One of the most notable findings was the thickening of the interalveolar septa due to proliferative changes and inflammatory cell infiltration. Inflammatory cell infiltration was also present within the interstitium, as well as in the vessels and their surrounding areas. Mild exudative changes associated with hyperemia were observed in the interstitial regions where proliferative inflammation predominated. Inflammation and associated exudation were nearly absent from the lumens of the bronchi, bronchioles, and alveoli; however, degenerative and necrotic lesions were noted within the bronchioles and alveoli. Mild fibromuscular hypertrophy was detected around the bronchioles and alveolar ducts, and lymphoid hyperplasia was observed in the peribronchial and peribronchiolar regions, and to a lesser extent around the alveoli. While atelectasis developed in some alveoli, extensive emphysematous areas resulting from rupture were observed in others following compensatory expansion (Figure 3A,B).

**Figure 4:**
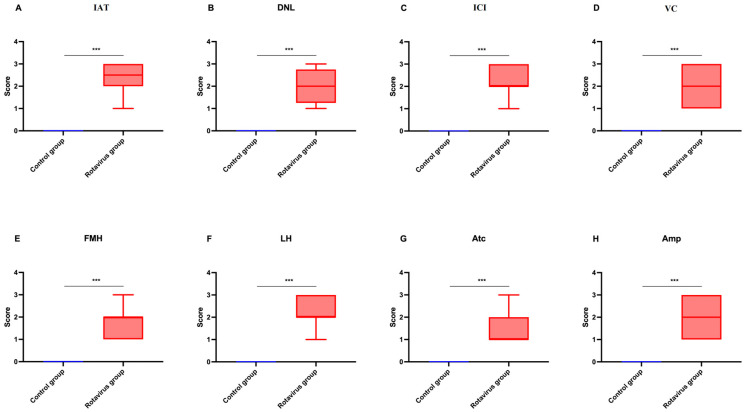
Graphical representation of histopathological scoring used for lung tissues: (**A**) Interalveolar thickening, (**B**) Degenerative-necrotic lesions, (**C**) Inflammatory cell infiltration, (**D**) Vascular changes, (**E**) Fibromuscular hypertrophy, (**F**) Lymphoid hyperplasia, (**G**) Atelectasis, (**H**) Emphysema. *** *p* < 0.001.

The statistical analysis of histopathological lesion severity scores in lung tissue from calves that died due to rotavirus infection is presented in Figure 4A–H.

**Figure 5:**
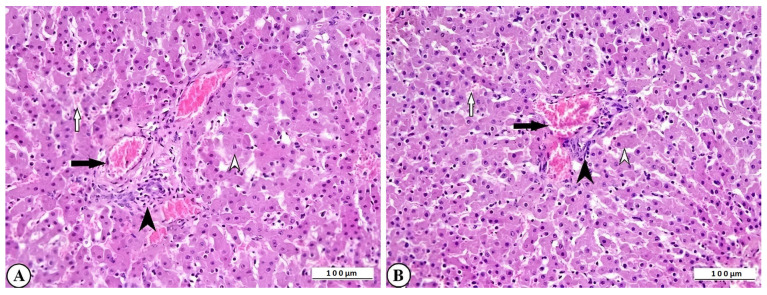
(**A**) Degenerative-necrotic changes in hepatocytes (open arrowhead), perivascular mononuclear leukocyte infiltration (arrowhead), vascular hyperemia (arrow), and hemorrhage in sinusoidal spaces (open arrow) in cattle liver, ×200, bar: 100 µm, HE. (**B**) Degenerative-necrotic changes in hepatocytes (open arrowhead), perivascular mononuclear leukocyte infiltration (arrowhead), vascular hyperemia (arrow), and hemorrhage in sinusoidal spaces (open arrow) of cattle liver, ×200, Bar: 100 µm, HE.

Severe histopathological alterations were observed in the liver tissue of rotavirus-infected calves. Hepatocytes exhibited hydropic degeneration and coagulative necrosis. These degenerative and necrotic lesions, which were most prominent in the central lobular region, disrupted the normal radial arrangement of hepatocytes. Vascular changes were characterized by marked hyperemia within the vessels and hemorrhages in the sinusoidal spaces. In addition, leukocyte infiltration, predominantly mononuclear cells, was evident, indicating an inflammatory response. These leukocytes, particularly those with perivascular localization, were also observed surrounding degenerative hepatocytes within the hepatic lamina (Figure 5A,B).

**Figure 6:**
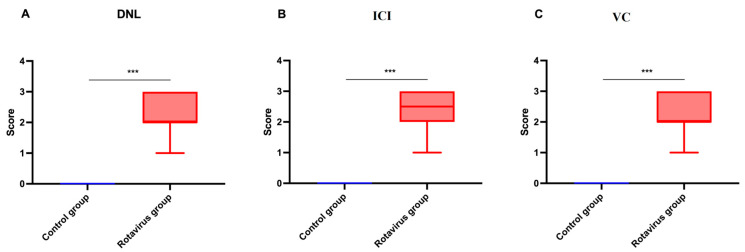
Graphical representation of histopathological scoring in liver tissue: (**A**) Degenerative-necrotic lesions, (**B**) Inflammatory cell infiltration, (**C**) Vascular changes. *** *p* < 0.001.

The statistical analysis of histopathological lesion severity scores in liver tissue from calves that died due to rotavirus infection is presented in Figure 6A–C.

### 2.5. Immunohistochemical Findings

**Figure 7:**
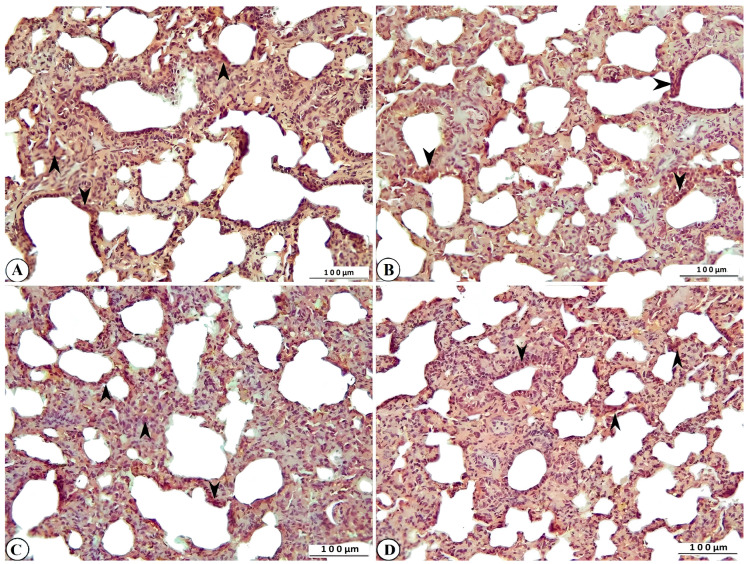
Immunohistochemical representation of (**A**) IL-2 (arrowhead), (**B**) HSP-27 (arrowhead), (**C**) CASP-3 (arrowhead), and (**D**) γ-H2AX expression (arrowhead) of cattle lung, ×200, Bar: 100 µm, IHC.

Immunohistochemical examination of rotavirus-infected lung and liver tissues evaluated inflammation using IL-2, oxidative stress using HSP-27, apoptosis using CASP-3, and DNA damage using γ-H2AX biomarker expressions. Varying levels of IL-2, HSP-27, CASP-3, and γ-H2AX expression were observed in the lung tissues of rotavirus-infected calves (Figure 7A–D).

**Figure 8:**
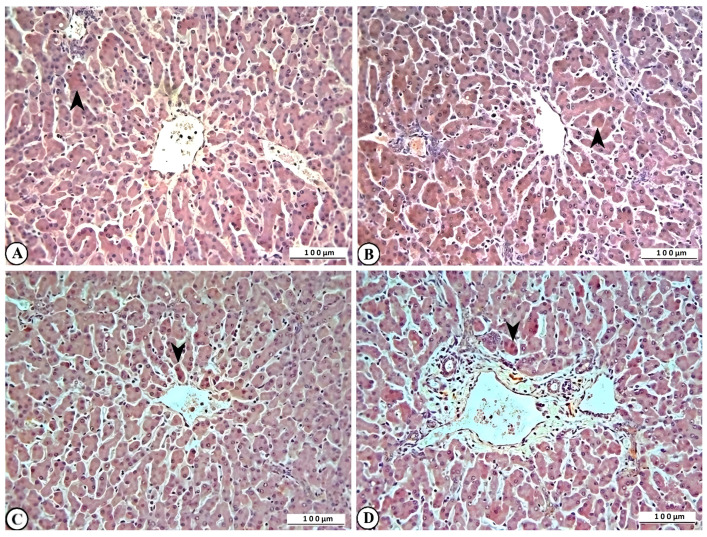
Immunohistochemical representation of (**A**) IL-2 (arrowhead), (**B**) HSP-27 (arrowhead), (**C**) CASP-3 (arrowhead), and (**D**) γ-H2AX expression (arrowhead) in bovine liver, ×200, Bar: 100 µm, IHC.

Marked expression of IL-2, HSP-27, CASP-3, and γ-H2AX was also observed in liver tissues of rotavirus-infected calves (Figure 8A–D).

**Figure 9:**
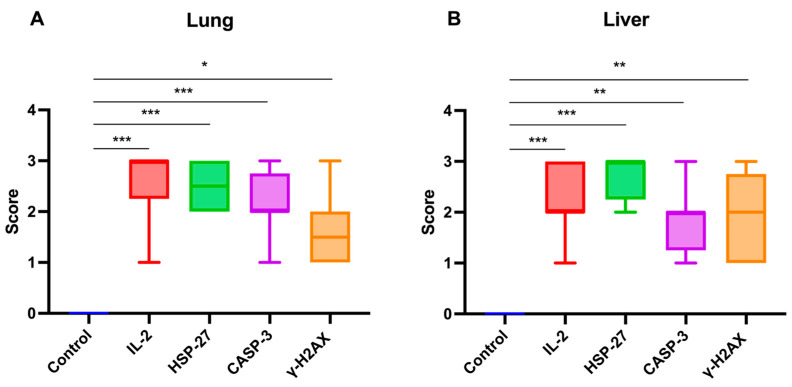
Graphical representation of immunohistochemical parameter expression intensities in (**A**) lung and (**B**) liver tissues of calves that died due to rotavirus infection. IL-2: Interleukin-2, HSP-27: Heat shock protein-27, CASP-3: Caspase-3, γ-H2AX: γ-phosphorylated form of histone *H2AX.* * *p <* 0.05, ** *p* < 0.01, *** *p* < 0.001.

The statistical analysis of immunohistochemical parameter expression intensities in lung and liver tissues from calves that died of rotavirus infection is presented in Figure 9A,B.

## 3. Discussion

Studies in human medicine have shown that rotavirus infection can also occur in extraintestinal sites (such as the lungs, liver, spleen, and brain) because it causes systemic effects [33,34,35,36]. However, in veterinary medicine, the injury caused by rotavirus diarrhea in calves has been considered limited to the intestines, and its effects on different systemic structures have not been fully evaluated [1,2,3,4,5]. This is the first study to assess extraintestinal injury in naturally infected calves with rotavirus using multiple techniques. In this study, we aimed to determine the effects of HSP-27, Caspase-3, IL-2, γ-H2AX, HMGB-1, SP-D, and GDH on the pathogenesis of rotavirus infection by using biomarkers of lung and liver injury in neonatal diarrheic calves naturally infected with rotavirus, both in vivo and postmortem. We found that serum GDH, SP-D, and HMGB-1 concentrations were significantly increased in rotavirus-infected calves, and Caspase-3, IL-2, HSP-27 and γ-H2AX expressions were intense in the lung and liver tissues. Both in vivo indicators (serum HMGB-1, SP-D, and GDH) and post-mortem findings (HSP-27, Caspase-3, IL-2, and γ-H2AX) demonstrated their involvement in rotavirus pathogenesis and in lung and liver injury. GDH is an enzyme involved in amino acid oxidation and urea production, primarily expressed in the liver [20,21,22,37]. It is much more sensitive to liver injury than ALT, and is better than ALT for detecting hepatocellular necrosis in rodents, and also shows a good correlation with liver injury [21]. Similarly, a study conducted in 843 subjects (364 healthy and 479 with varying degrees of liver injury) found that GDH had high diagnostic power for detecting liver injury [38]. Comparable accuracy was observed in dogs, where serum GDH concentrations more reliably indicated the absence of liver injury after treatment than ALT concentrations [22]. In agreement with previous studies, our study confirmed the predictive value of serum GDH concentrations in rotavirus-infected calves. Liver histopathology also revealed hepatocyte degeneration, coagulation necrosis, and vascular changes in the form of severe hyperemia and hemorrhage in the sinusoidal spaces. In this study, liver damage may be related to rotavirus’s systemic effects on calves, spreading to extraintestinal tissues and causing apoptosis, oxidative stress, and inflammation. HMGB-1 is known to play roles in DNA binding and regulation [20,39]. However, recent studies have revealed that it is released into the circulation from injured or necrotic liver cells [20,40,41]. Serum HMGB-1 concentration increased earlier and more rapidly than ALT when liver injury was experimentally induced in mice, and showed a strong correlation with serial liver histopathological evaluations [41]. In parallel, another study demonstrated that HMGB-1 was more sensitive than ALT in determining hepatotoxicity [33]. Based on these findings, a therapeutic study using chimeric humanized anti-HMGB-1 antibodies reported improvements in acetonitrile-induced liver injury in mice [40]. Therefore, HMGB-1 plays a significant role in liver injury and can be used to detect it. The significantly higher HMGB-1 concentrations in rotavirus-infected calves in this study, compared to the control group, are consistent with previous studies. Additionally, ROC analysis demonstrated good diagnostic performance (AUC: 0.89; sensitivity: 80%; specificity: 80%) for detecting liver injury. Consistent with the concept that HMGB-1 leakage from injured or necrotic cells stimulates its active release from inflammatory and immune cells [20,35], we also found that IL-2, an important inflammatory cytokine, was expressed in liver tissue simultaneously with increased serum HMGB-1 concentrations. Due to the release of SP-D from alveolar type II epithelial cells, many studies in recent years have investigated the diagnostic, prognostic, and therapeutic application of this biomarker in various lung diseases [14,15,16,17]. Studies conducted on patients with acute respiratory distress syndrome revealed that plasma SP-D concentrations were significantly elevated and could be used diagnostically to identify the syndrome [14,15]. Conversely, SP-D concentration has been considered a valuable predictor of disease severity and clinical outcomes in patients with acute lung injury [17]. Similarly, circulating SP-D concentration in patients infected with the A/H1N1 virus was associated with a higher risk of mortality and was used to predict poor outcomes in viral pneumonia [16]. Consistent with previous data, this study also found that SP-D concentration was significantly increased in rotavirus-infected calves and demonstrated excellent diagnostic performance (AUC: 0.91, sensitivity: 80%, specificity: 93.3%) in distinguishing rotavirus-infected calves from uninfected controls. To the best of our knowledge, this study is the first to demonstrate that HSP-27, Caspase-3, IL-2, and γ-H2AX are expressed in the lung and liver tissues of calves naturally infected with rotavirus, indicating that oxidative stress, apoptosis, and DNA damage may underlie rotavirus infection. For instance, γ-H2AX plays a pivotal role in DNA repair [28,34], and its overexpression has been observed in immunohistochemical analyses of patients with non-squamous lung cancer, suggesting that this biomarker is a prognostic indicator of disease progression [27]. In patients with hepatocellular carcinoma, increased γ-H2AX positivity has also been observed in liver biopsies, indicating significant injury. Although an in vitro study showed that rotaviruses can cause DNA fragmentation through nuclear apoptosis [38], no in vivo studies have previously demonstrated DNA double-strand breaks caused by rotaviruses via histone phosphorylation. In this study, immunopathological analyses of lung and liver tissues in rotavirus-infected calves revealed γ-H2AX overexpression.

IL-2 is a proinflammatory cytokine produced by T helper cells and involved in the activation and proliferation of most T lymphocytes [29,35]. In pigs naturally infected with *Mycoplasma hyopneumoniae*, intense immunohistochemical IL-2 expression was reported in the bronchus-associated lymphoid tissues of the lungs, contributing to cellular and humoral immune responses [29]. Kasprzak et al. (2004) also reported increased IL-2 expression in patients with chronic hepatitis [42].

Crawford et al. (2006) reported that experimental rotavirus infection in rats induced inflammation in extraintestinal organs, particularly the liver and lungs, as demonstrated by histopathological examination [43]. However, no studies have previously reported histopathological or immunopathological evidence of inflammation in the lungs and liver during rotavirus infection. This study demonstrated histopathologically that rotavirus causes inflammation in lung and liver tissues, further supported by the expression of IL-2, a major proinflammatory cytokine.

Caspase-3 activation has been reported to be increased in the liver lobules of patients with chronic hepatitis C virus infection compared with normal controls, and the degree of its activation correlated significantly with the extent of disease, including necroinflammatory activity [28]. Similarly, in patients with hepatocellular carcinoma, Caspase-3 was overexpressed and suggested to have diagnostic and therapeutic value [34]. Conversely, no studies have reported apoptosis in lung or liver tissues during natural rotavirus infection.

HSP-27 is involved in stress conditions responses, including hypoxia [44], oxidative stress [45], ischemia [44], and elevated temperature [44], and is known to be expressed in various diseases (e.g., hepatocellular carcinoma [30]). Interestingly, although no studies have demonstrated oxidative stress involvement in lung and liver tissues during natural rotavirus infection, we report for the first time that HSP-27 expression, alongside IL-2 and Caspase-3, may serve as a predictive biomarker in lung and liver tissues of calves naturally infected with rotavirus [30].

E-selectin, a member of the selectin family, is an endothelial molecule characterized by its N-terminal determinants and by its ability to bind sialylated glycan ligands. This molecule interacts with various ligands and contributes to both acute and chronic inflammation, offering potential therapeutic opportunities for multiple diseases [46]. In their study on calves with perinatal asphyxia, Ider et al. (2022) reported that E-selectin concentration was elevated and may be a useful biomarker for predicting mortality, with a cut-off value of 2.71 ng/mL, 70% sensitivity, and 60% specificity [47]. Similarly, in a study in which dogs were experimentally induced with LPS, E-selectin concentrations were also increased [48]. In contrast, Unal et al. (2025), in a study of calves with rotavirus diarrhea, observed no statistically significant difference in E-selectin concentrations between infected and control groups [49], findings consistent with our study. Differences between this study and other studies may reflect variations in disease type, biological and analytical factors, and disease severity.

This study has some limitations. First, the relationship between metabolites such as D-lactate and L-lactate and lung and liver injury requires further investigation. Although approximately 42.85% of calves in this study with rotavirus infection died, studies including larger numbers of fatal cases are needed to provide a comprehensive understanding.

## 4. Materials and Methods

### 4.1. Study Design and Grouping

The experimental design of this study was approved by the Bingöl University Animal Experiments Local Ethics Committee (B.Ü. HADYEK, Date: 2025/04, Decision No: 04/10) before the research began. All procedures were carried out in accordance with the relevant guidelines and regulations, as well as the Animal Research: Reporting of In Vivo Experiments (ARRIVE) guidelines. This is an observational case–control study on a total of 52 Simmental calves (between 1 and 28 days of age) of both sexes (male and female). Forty out of 52 calves were rotavirus-infected (21 males, 19 females) and 12 were healthy (6 males, 6 females). Calves naturally infected with rotavirus were obtained from a medium-sized farm in Bingöl province. Calves from other farms were not selected. The minimum required sample size was determined using G*power version 3.1.9.7 based on WBC count data. Using data obtained from a previous study on rotavirus-infected calves [1], the sample size calculation included and effect size of 0.9433086, a significance level of 0.05, and a statistical power of 95%.

### 4.2. Healthy Calves Group

Twelve healthy calves in the control group underwent hematological, biochemical, and blood gas analyses. Calves within values within reference ranges were considered healthy. Additionally, the following groups were excluded from the study: individuals who tested positive on a rapid fecal antigen test; those with congenital or acquired diseases or anomalies; those who had received antibiotics or vaccinations; those not between 1 and 28 years of age; and calves of other breeds. Healthy calves received colostrum at least 10% of their live weight during each of the first two days of life, and they were fed milk twice a day using individual feeding bottles.

### 4.3. Rotavirus-Infected Calves Group

Forty calves naturally infected with rotavirus were enrolled in this study. Twenty-eight out of 40 survived and the remaining 12 were used for histological and immunohistochemical analysis. Immunochromatographic rapid test kits (Anigen Rapid BoviD-5 Ag Test Kit, Bionote, Inc., Hwaseong-si, Republic of Korea) were used to detect rotavirus in calves. These kits provide rapid, sensitive field-based diagnosis, eliminating the need for laboratory testing [50,51]. According to the manufacturer, the polymerase chain reaction sensitivity and specificity of the test for rotavirus are 99% and 98%, respectively. Only rotavirus-positive samples were included in the study. Once the infection was confirmed, the calves were monitored at regular intervals (every 7 days until day 28). Calves that survived beyond day 28 were classified as survivors, whereas those that died before day 28 were classified non-survivors. Non-surviving calves underwent necropsy for histopathological and immunopathological examination of the lung and liver.

### 4.4. Sample Collection and Laboratory Analysis

Blood samples from the rotavirus and control groups were collected once from the jugular vein for hematological, biochemical, and blood gas analyses, using tubes with and without anticoagulants and lithium heparin syringes, in accordance with standard procedures. Blood samples were collected anaerobically in 2 mL lithium heparin syringes. For biochemical analyses, blood was collected in tubes without anticoagulant (BD Vacutainer^®^, Plymouth, UK). These samples were allowed to clot at room temperature for 1 h and then centrifuged at 5000 rpm for 5 min to obtain serum. Serum samples were then aliquoted into 0.5 mL portions and stored at −20 °C until analysis.

EDTA blood samples were collected from each calf using microhematocrit capillary tubes (Marienfeld, Germany) and centrifuged at 14,000× *g* for 5 min to determine the hematocrit value. Manual WBC counting was performed using a Thoma slide. Blood gas and electrolyte analyses were conducted using an automatic blood gas analyzer (Wondko Veterinary Blood Gas Analyzer Vcare-5000, Shenzhen, China). Serum analyses for aspartate aminotransferase, alanine aminotransferase, gamma-glutamyl transferase (GGT), urea, creatinine, total protein, and albumin were performed using an automatic biochemistry analyzer (Mindray BS-2000m, Shenzhen, China). Serum E-selectin (Bovine E-selectin ELISA kit^®^, Shanghai Coon Koon Biotech Co., Ltd., Shanghai, China, Catalog No: CK-bio-25334), GDH (Bovine Glutamate Dehydrogenase ELISA kit^®^, Shanghai Coon Koon Biotech Co., Ltd., China, Shanghai, Catalog No: CK-bio-26401), SP-D (Bovine Pulmonary Surfactant-Associated Protein D ELISA kit^®^, Shanghai Coon Koon Biotech Co., Ltd., Shanghai, China, Catalog No: CK-bio-26015), and HMGB-1 (Bovine High Mobility Group Protein B1 ELISA kit^®^, Shanghai Coon Koon Biotech Co., Ltd., Shanghai, China, Catalog No: CK-bio-26016) were analyzed by means of a bovine-specific ELISA kit. The ELISA procedures were performed according to the manufacturer’s instructions and the optical density was measured with a microplate reader (BioTek Instruments^®^, Winooski, VT, USA) at 450 nm. Standards were analyzed in duplicate to improve reliability. For all ELISA kits the intra-assay coefficient of variation (CV) was <7% and inter-assay CV was <10%.

### 4.5. Histopathological Examination

After necropsy, tissue samples were fixed in 10% buffered formaldehyde. Following fixation, tissues were washed under running tap water using routine tissue-monitoring procedures. Each sample was embedded in paraffin, and 5 µm-thick sections were cut onto normal and adhesive slides using a rotary microtome (Leica RM 2125, Schönwalde-Glien, Germany). The sections placed on normal slides were heated in an oven, deparaffinized and rehydrated. Two investigators blindly evaluated six different microscopic fields at 20× magnification for each specimen. Hematoxylin–eosin-stained sections were coverslipped using Entellan^TM^ and examined under a light microscope (Olympus BX53, Tokyo, Japan) [36]. An overall histological score was assigned to each rotavirus-infected tissue based on semi-quantitative criteria, adapted from Canelli et al. 2023 [52], including DNL, ICI, VC, FMH, LH, Amp, Atc, IL-2, HSP-27, CASP-3, and γ-H2AX [53,54] (Table 4).

### 4.6. Immunohistochemical Examination

Tissue sections mounted on adhesive slides were first warmed in an oven, followed deparaffinization and rehydration. The sections were then incubated in 3% H_2_O_2_ for 10 min to inactivate endogenous peroxidase activity and subsequently washed with phosphate-buffered saline (PBS).

Antigen retrieval was performed by boiling and cooling the sections in an antigen retrieval solution, followed by another PBS wash. To prevent non-specific binding, sections were incubated with a protein block within PAP-pen-limited areas. Primary antibodies against HSP-27, Caspase-3, IL-2, and γ-H2AX were diluted 1:100 and applied to the sections for overnight incubation at 4 °C. After washing with PBS, a biotinylated secondary antibody compatible with the primary antibodies was applied, followed by another PBS wash. Streptavidin-peroxidase was then added for incubation, and the sections were washed again with PBS. Mayer’s hematoxylin was used as a counterstain to visualize antigen–antibody binding. The slides were coverslipped and examined under a light microscope (Olympus BX 53, Tokyo, Japan). Two blinded investigators evaluated immunostaining, and immunopositivity was assessed semi-quantitatively [55]. The percentage of positive cells was scored on a 5-point scale: 0 = no positive cells, 1 ≤ 20%, 2 = 21–50%, 3 = 51–70%, and 4 ≥ 71%. Immunopositive and immunonegative cells were counted in each microscopic field and converted to percentages. Staining intensity was scored on a 4-point scale: 0 = no staining, 1 = low intensity, 2 = moderate intensity, and 3 = high intensity. For samples with heterogeneous intensity, the chosen score was the predominant one within each sample.

### 4.7. Statistical Analysis

Data were analyzed using SPSS 26 (IBM SPSS Statistics for Windows, Version 22.0. Armonk, NY, USA: IBM Corp.) and GraphPad Prism (Prism 9 for Windows, version 9, (GraphPad Software, Inc., La Jolla, CA, USA). The data of the study were given as mean ± standard deviation, minimum, and maximum. Homogeneity of variances was assessed with the Levene’s test. Data normality was evaluated using the Shapiro–Wilk Test. The Mann–Whitney test was applied to evaluate differences among DNL, ICI, VC, FMH, LH, Amp, and Atc for liver and lung histopathological scoring in rotavirus-infected calves [53,56]. A Kruskal–Wallis test followed by Dunn’s multiple comparisons test was used to evaluate differences among IL-2, HSP-27, CASP-3, and γ-H2AX for liver and lung immunohistochemical scoring in rotavirus-infected calves.

ROC curve analysis was performed to determine sensitivity, specificity, and cut-off values. The relationship between variables was determined using the Spearman correlation coefficient test. The values of the correlation coefficients were interpreted as follows: r = 0.00–0.10, negligible correlation; r = 0.10–0.39, weak correlation; r = 0.40–0.69, moderate correlation; r = 0.70–0.89, strong correlation; r = 0.90–1.00, powerful correlation [57]. A *p* < 0.05 was considered significant.

## 5. Conclusions

In conclusion, this study demonstrated lung and liver injury in rotavirus-infected calves both in vivo (HMGB-1, SP-D, and GDH) and post-mortem (HSP-27, CASP-3, γ-H2AX, and IL-2), along with severe histological tissue destruction driven by inflammatory responses. Immunopathological examination revealed IL-2 expression, indicating inflammation in these organs. Collectively, these findings suggest that oxidative stress (HSP-27), apoptosis (CASP-3), and DNA damage (γ-H2AX) may contribute the pathogenesis of this condition. However, further studies on large populations of rotavirus-infected, non-surviving calves are needed to confirm the lung and liver injury observed in the current study.

## Figures and Tables

**Table 1:** Concentrations of biochemical values and clinical examination findings in rotavirus-infected calves.

Variables	Rotavirus Group	Control Group	*p* Value
Heart Rate (beats/minute)	136 (100–180)	125 (92–160)	0.751
Respiratory Frequency (respiratory rate/minute)	34.66 (20–60) ^a^	45 (30–56) ^b^	<0.05
Body Temperature (°C)	38.2 (35.5–40.4)	38 (38.4–39.2)	0.117
HCT (%)	34.33 (24–47)	32.7 (24.2–40)	0.235
WBC (×10^9^)	18.64 (5.1–43.44) ^a^	10.2 (6.43–13.9) ^b^	<0.001
Blood pH	7.13 (6.69–7.44) ^a^	7.36 (7.28–7.4) ^b^	<0.001
Bicarbonate (mmol/L)	13.94 (7.1–25.2) ^a^	26.41 (19.7–29.8) ^b^	<0.001
Glucose (mmol/L)	4.01 (1–7.6) ^a^	5.09 (3.7–6.1) ^b^	<0.05
Calcium (mmol/L)	1.09 (0.63–1.33) ^a^	1.4 (1.37–1.55) ^b^	<0.001
Sodium (mmol/L)	131.69 (113–146)	139 (125–141)	0.133
Potassium (mmol/L)	5.55 (3.5–8.4) ^a^	4.65 (4.2–5.1) ^b^	<0.05
Chlorine (mmol/L)	105.8 (81–140)	95 (89–101)	0.100
Total protein (mg/dL)	8.47 (6.39–10.32)	8.84 (6.61–9.81)	0.126
Albumin (g/L)	3.85 (3.16–4.49)	4.07 (2.78–4.75)	0.180
Creatin (mmol/L)	1.57 (1.1–2.31)	1.4 (1.2–1.67)	0.229
BUN (mg/dL)	40.56 (8.07–91.27)	33.13 (20.02–42.21)	0.333
ALT (U/L)	18.04 (6.16–60) ^a^	8.32 (6.11–12.14) ^b^	<0.01
AST (U/L)	87.49 (29.62–491.38) ^a^	41.38 (32.85–70.35) ^b^	<0.05
GGT (U/L)	210.19 (17–2691)	73.76 (10.87–153.9)	0.126

ALT, alanine aminotransferase; AST, aspartate aminotransferase; GGT, gamma-glutamyl transferase; HCT, hematocrit; WBC, White blood cell; BUN, Blood urea nitrogen. ^a,b^: The differences between the groups with different letters on the same line are significant (*p* < 0.05).

**Table 2:** Serum biomarker concentrations in rotavirus-infected calves are shown.

Variables	Rotavirus Group	Control Group	*p* Value
E-selectin (pg/mL)	179.52 (76.55–260.86)	173.5 (131.82–197.77)	0.803
HMGB-1 (pg/mL)	682.76 (154–1028.2) ^a^	477.94 (348–612) ^b^	<0.001
SP-D (pg/mL)	2.32 (1.13–3.15) ^a^	1.36 (0.8–2.19) ^b^	<0.001
GDH (U/L)	1.39 (0.7–2.51) ^a^	1.09 (0.8–1.5) ^b^	<0.05

HMGB-1: high mobility group box, SP-D: surfactant protein-D, GDH: glutamate dehydrogenase. ^a,b^: The differences between the groups with different letters on the same line are significant. *p* < 0.05.

**Table 3:** Lung and liver biomarkers in rotavirus-infected calves.

Variables	AUC	Sensitivity	Specificity	Cut-Off Point	*p* Value
SP-D (pg/mL)	0.91	80	93	1.955	<0.001
GDH (U/L)	0.74	70	70	1.185	<0.05
HMGB-1 (pg/mL)	0.89	80	80	518.8	<0.01

HMGB-1: high mobility group box, SP-D: surfactant protein-D, GDH: glutamate dehydrogenase, AUC: area under the curve.

**Table 4:** Evaluation of pathological scoring parameters of rotavirus-infected tissues.

Histological Feature	Score	Description
Degenerative-necrotic lesions	0	none
	1	minimal
	2	mild
	3	moderate
	4	severe
Inflammatory cell infiltration	0	none
	1	minimal
	2	mild
	3	moderate
	4	severe
Vascular changes	0	none
	1	minimal
	2	mild
	3	moderate
	4	severe
Emphysema	0	none
	1	minimal
	2	mild
	3	moderate
	4	severe
Interalveolar thickening	0	none
	1	minimal
	2	mild
	3	moderate
	4	severe
Lymphoid hyperplasia	0	none
	1	minimal
	2	mild
	3	moderate
	4	severe
Fibromuscular hypertrophy	0	none
	1	minimal
	2	mild
	3	moderate
	4	severe
Atelectasis	0	none
	1	minimal
	2	mild
	3	moderate
	4	severe
IL-2	0	none
	1	minimal
	2	mild
	3	moderate
	4	severe
HSP-27	0	none
	1	minimal
	2	mild
	3	moderate
	4	severe
CASP-3	0	none
	1	minimal
	2	mild
	3	moderate
	4	severe
γ-H2AX	0	none
	1	minimal
	2	mild
	3	moderate
	4	severe

## Data Availability

The original contributions presented in this study are included in the article. Further inquiries can be directed to the corresponding author.

## Abbreviations

The following abbreviations are used in this manuscript:

HMGB-1	High mobility group box
SP-D	Surfactant Protein D
GDH	Glutamate dehydrogenase
ARDS	Acute respiratory distress syndrome
ALT	Alanine aminotransferase
γ-H2AX	γ-phosphorylated form of histone *H2AX*
IL-2	Interleukin-2
AST	Aspartate aminotransferase
GGT	Gamma-glutamyl transferase
HCT	Hematocrit
WBC	White blood cell
BUN	Blood urea nitrogen
AUC	Area under the curve
HSP-27	Heat shock protein-27
CASP-3	Caspase-3
ROC	Receiver-operating characteristic curve
PBS	Phosphate-buffered saline
